# Obstructive sleep apnea in patients with acute aortic dissection

**DOI:** 10.1002/clc.23790

**Published:** 2022-02-23

**Authors:** Evan J. Friend, Pavel Leinveber, Marek Orban, John Hochhold, Anna Svatikova, Virend K. Somers, Gregg S. Pressman

**Affiliations:** ^1^ Division of Cardiology, Institute for Heart and Vascular Disease Einstein Medical Center Philadelphia Philadelphia Pennsylvania USA; ^2^ International Clinical Research Center, St. Anne's University Hospital Brno Czech Republic; ^3^ Comenius University and NUSCH Bratislava Slovakia; ^4^ Department of Cardiovascular Medicine Mayo Clinic Rochester Minnesota USA

**Keywords:** aortic diameter, aortic dissection, obstructive sleep apnea, modified Berlin Questionnaire

## Abstract

**Background:**

Obstructive sleep apnea (OSA) imposes an afterload burden on the left ventricle and increases the pressure gradient across the aortic wall. Thus, OSA may increase the risk for aortic dissection (AD).

**Methods:**

This study enrolled 40 subjects with acute AD from four institutions; 37 completed the modified Berlin Questionnaire and 31 underwent attended overnight polysomnography. Aortic diameter was measured on a computed tomography scan at seven locations from the sinotubular junction to the diaphragm.

**Results:**

Twenty‐seven subjects had type A dissection; 13 had type B. In those who had polysomnography apnea–hypopnea index (AHI) ranged from 0.7 to 89. Prevalence of OSA (AHI ≥ 5) was 61%. Nocturnal presentation (10 p.m.–7 a.m.) did not differ by presence/absence of OSA. The modified Berlin Questionnaire was not predictive of the presence of OSA. Among type A subjects with polysomnography (*n* = 23), aortic diameters at all locations were greater in the OSA group though differences were not statistically significant. Summating aortic diameters at the seven locations also yielded a numerically larger mean value in the OSA group versus the non‐OSA group.

**Conclusions:**

In this sample of patients with acute dissection, OSA was prevalent but was not associated with a nocturnal presentation. The presence of underlying OSA may be associated with larger aortic diameters at the time of dissection compared to patients without OSA. Though differences did not meet statistical significance the current series is limited by small numbers.

## 1 BACKGROUND

Obstructive sleep apnea (OSA) imposes an afterload burden on the left ventricle^1^ and increases the pressure gradient across the intrathoracic aorta (as a consequence of the Mueller Maneuver—forced inspiration against an occluded airway).^2^ Given that obstructive apneas can occur hundreds of times per night in patients with severe OSA, the intrathoracic aorta is exposed to repeated episodes of sudden changes in wall stress. This might increase the likelihood of aortic dissection (AD). Pressor and hypoxic stressors resulting from obstructive apneas have been implicated in the increased risk of other acute cardiovascular events, including myocardial infarction, ventricular arrhythmias, and sudden death, especially when they occur at night.^3^, ^4^, ^5^ We, therefore, hypothesized that patients presenting with acute AD would have a high prevalence of undiagnosed OSA and a largely nocturnal presentation. We further examined the utility of a questionnaire‐based assessment (the modified Berlin Questionnaire) to evaluate AD patients for the presence of OSA.

## 2 METHODS

Forty consecutive consenting subjects with acute AD who survived to hospital discharge were prospectively enrolled from four institutions (Einstein Medical Center Philadelphia; Thomas Jefferson University Hospital, Philadelphia; Center for Cardiovascular and Transplantation Surgery, Brno, Czech Republic; Mayo Clinic, Rochester, MN). Informed consent was signed by each patient and the study protocol was approved by the Institutional Review Board or Ethics Committee at each of the respective institutions.

Thirty‐seven subjects completed the modified Berlin Questionnaire, a survey designed to identify patients at high risk of OSA. Thirty‐one subjects had attended overnight polysomnography (PSG), the gold standard test for diagnosis of OSA. Three of these subjects had undergone sleep testing before experiencing their dissection; the others had an elective PSG performed within a year of discharge from the hospital. Scoring was done according to American Academy of Sleep Medicine standards using the 3% hypopnea rule (hypopneas defined by ≥ 3% oxygen desaturation from pre‐event baseline). Presence or absence of OSA was based on an apnea–hypopnea index (AHI) ≥ 5/h with cutoffs of > 15 and > 30 used to define moderate and severe OSA.

Computed tomography (CT) scans on presentation were available in all subjects. Type A dissection included those where the dissection originated before the origin of the left subclavian artery while for type B the origin of the dissection was distal to the left subclavian. Aortic diameter was measured at seven locations within the thoracic cavity: (1) the sinotubular junction, (2) ascending aorta, (3) origin of the innominate artery, (4) mid‐arch, (5) origin of the left subclavian artery, (6) mid‐descending aorta, and (7) level of the diaphragm. Follow‐up CT scans at 3 months were available in 24 subjects. Diameters were measured from outer wall to outer wall of the aorta by either one of two cardiothoracic radiologists, each with more than 10 years of experience. In aortic segments with dissection, both the true lumen and the false lumen were included in the diameter measurements. Measurements were made from whichever plane (axial, coronal, or sagittal) would allow for the most accurate orthogonal depiction of an aortic segment, as judged by the radiologist. Interobserver variability was not assessed.

Categorical variables are presented as number and percentage and continuous variables as mean ± SD. Comparisons between categorical variables were done using either the chi‐squared test or the Fisher exact test, as appropriate. For continuous variables, the Student *t* test or the Wilcoxon test, when values were not normally distributed, was used. A two‐tailed *p* < .05 was considered to indicate statistical significance. Statistical analyses were performed using JMP version 14.0 (SAS Institute).

## 3 RESULTS

Patient characteristics are found in Table 1. Mean age was 57 (range 28–75), 24 subjects were male. 27 had type A dissection while 13 had type B. Hypertension was very prevalent (30/40, 75%) while diabetes was less so (5/40, 12.5%). Two subjects had a history of myocardial infarction of whom one had undergone prior CABG. Smoking was present in two of the 31 subjects for whom this information was available. Comparing type A with type B dissections, aortic diameters were greater from the sinotubular juncture through and including the left subclavian location in type A dissection. Conversely, aortic diameters distal to the left subclavian were similar between the two types of dissection (Table 2). From the index CT scan to one done 3 months later, aortic diameter decreased at the level of the sinotubular junction and ascending aorta (expected—due to surgery for type A dissections) with no other significant changes noted.

**Table 1:** Patient characteristics

	*N* = 40
Age (years)	56.6 ± 11.3
Male (%)	24 (60)
BMI	32.4 ± 10.6
HTN (%)	30 (75)
DM (%)	5 (12.5)
CAD (%)	2 (5)

Abbreviations: BMI, body mass index; CAD, coronary artery disease; DM, diabetes mellitus; HTN, hypertension.

**Table 2:** Aortic diameters on initial CT scan by dissection type

	STJ	Asc Ao	Innom	Mid‐Arch	L Subcl	Desc Ao	Diaphragm
All subjects	3.6 ± 0.9	4.2 ± 1.0	3.4 ± 0.7	3.3 ± 0.7	3.0 ± 0.5	3.2 ± 0.5	3.0 ± 0.4
Type A (*n* = 27)	4.0 ± 0.9	4.6 ± 0.9	3.7 ± 0.6	3.5 ± 0.7	3.1 ± 0.6	3.2 ± 0.4	2.9 ± 0.3
Type B (*n* = 13)	3.1 ± 0.7	3.6 ± 0.7	3.1 ± 0.6	3.0 ± 0.5	2.8 ± 0.4	3.2 ± 0.6	3.0 ± 0.6
Type A subjects with PSG							
AHI < 5 (*n* = 9)	3.9 ± 0.7	4.4 ± 0.8	3.5 ± 0.5	3.3 ± 0.8	3.0 ± 0.6	3.1 ± 0.5	2.8 ± 0.3
AHI ≥ 5 (*n* = 14)	4.1 ± 1.1	4.7 ± 1.1	3.8 ± 0.7	3.6 ± 0.8	3.2 ± 0.6	3.3 ± 0.3	3.0 ± 0.3

*Note*: All measurements in cm.

Abbreviations: AHI, apnea‐hypopnea index; Asc Ao, ascending aorta; CT, computed tomography; Desc Ao, mid descending aorta; Diaphragm, at the level of the diaphragm; Innom, at the level of the innominate; L Subcl, at the level of the left subclavian; PSG, polysomnography; STJ, sinotubular junction.

In those who had formal sleep testing (*n* = 31), AHI ranged from 0.7 to 89. Prevalence of OSA (defined by AHI ≥ 5) was 61% (*n* = 19); 42% (*n* = 13) had AHI ≥ 15%; and 29% (*n* = 9) had AHI ≥ 30. Using AHI ≥ 5 to define the presence of OSA both body‐surface area (2.28 ± 0.08 vs. 1.97 ± 0.29, *p* = .02) and body‐mass index (36.8 ± 12.8 vs. 28.6 ± 6.5, *p* = .06) were greater in the OSA group versus the non‐OSA group. Whether dissection occurred during overnight hours (10 p.m.–7 a.m.) did not differ by presence/absence of OSA (regardless of AHI cutoff). AHI was not significantly different between those with high‐risk Berlin questionnaires (≥ 2 categories positive) and those without, thus the questionnaire was not useful in diagnosing the presence of OSA. Distribution of high‐risk Berlin Questionnaires was similar among dissection types. Among the 37 who completed the questionnaire 11 (30%) felt fatigued during waking hours, 13 (35%) felt fatigued after sleep, and 8 (22%) had nodded off while driving.

**Figure 1:**
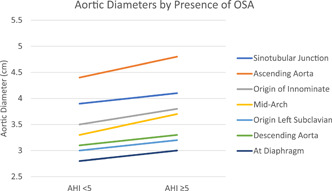
Aortic diameters by the presence of obstructive sleep apnea (OSA). Each line compares the mean aortic diameter between those without OSA (apnea–hypopnea index [AHI] < 5, left) and those with OSA (AHI ≥ 5, right) at each of seven points along the aorta. Note that at each point the aortic diameter is greater in those with OSA vs. those without

**Figure 2:**
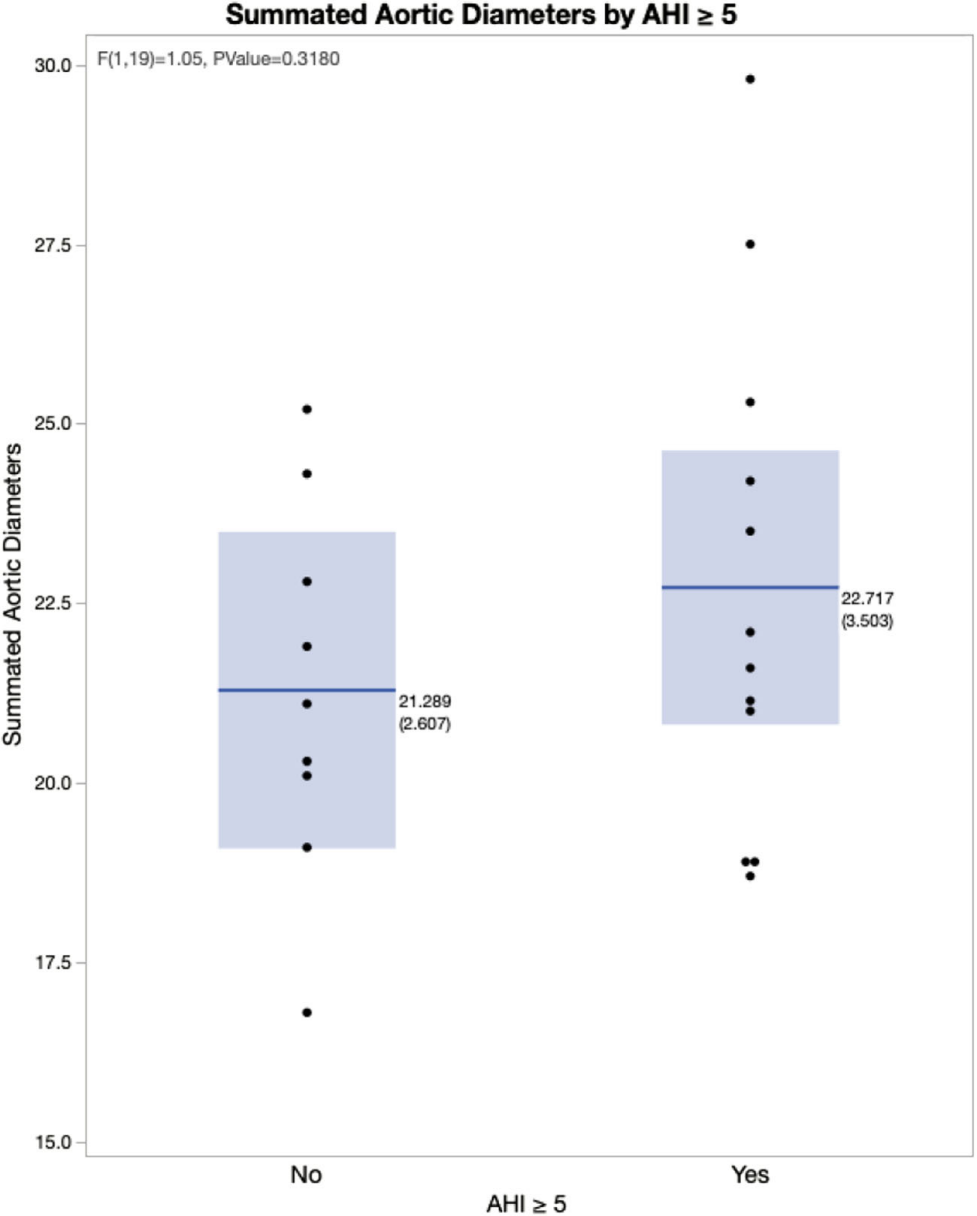
Summated aortic diameters by the presence of obstructive sleep apnea (OSA). For this plot, the individual aortic diameters at each of the seven measurement points were summated for each patient. The box plots display the median value, and 25th and 75th percentiles, for the group without OSA (apnea–hypopnea index [AHI] < 5, left) and the group with OSA (AHI ≥ 5, right). The dots represent individual subjects

We next focused on the 23 subjects with type A dissection who had a PSG. Aortic diameters at the various levels were compared between those with OSA (AHI ≥ 5, *n* = 14) and those without OSA (AHI < 5, *n* = 9; Figure 1 and Table 2). At all locations mean aortic diameters were greater in the OSA group versus the non‐OSA group though differences did not reach statistical significance. We then summated the aortic diameters and compared the two groups. Again we found numerically larger values in the OSA group (Figure 2). Due to the small number of subjects the findings did not meet statistical significance. There were too few type B dissections to make the same comparisons.

## 4 DISCUSSION

Obstructive sleep apnea is associated with the presence and growth of thoracic aortic aneurysms.^6^, ^7^ Several studies have suggested that OSA increases the risk for aortic complications^6^, ^8^, ^9^ but prospective studies are rare. The current research reveals several interesting observations. First, that patients with acute AD have a high prevalence of OSA and that many subjects had moderate or severe OSA (AHI > 15). Though this prevalence is similar to that of other reports^9^, ^10^ it is higher than might be expected in a group with a median BMI of only 28. Additionally, we had hypothesized that OSA subjects who develop AD would have a nocturnal presentation. That was not the case in this sample though it is still possible that a larger study would find such an association.

Second, we found no evidence that the modified Berlin questionnaire was useful in identifying the presence of OSA in our patients. This and other questionnaires have acknowledged limitations.^11^, ^12^ A rapid screening tool for the presence of OSA is an unmet need.

The third observation of interest is that of larger aortic diameters at the time of dissection in patients with OSA. We focused on the subgroup with type A dissection as there were only eight type B dissections among those who had a formal sleep study. While differences in aortic diameter between OSA and non‐OSA patients were not statistically significant (likely due to small numbers) it is notable that at each of the seven‐points aortic diameters were numerically greater in the sleep apnea group. Summating the aortic diameters also yielded numerically higher values in these patients, suggesting that the aorta was affected diffusely rather than segmentally. This may be a signal that subjects with OSA are not only more prone to dissection but have larger aortic diameters as a result of the dissection.

Given the repetitive nature of obstructive apneas and associated wall stress on the aorta, one might expect OSA subjects to accumulate greater degrees of aortic damage than non‐OSA subjects. Indeed, others have observed larger diameters of the aortic root/ascending aorta in subjects with OSA.^13^, ^14^ Here, we report larger aortic dimensions measured immediately after occurrence of dissection. Thus, OSA patients may also experience worse effects of dissection than those without OSA.

It is still possible that obstructive apnea itself is not an independent risk factor for AD. It may be the case that wall shear stress (force parallel to the aortic wall) is a more important factor in AD^15^ than wall stress perpendicular to the aorta (such as that imposed by obstructive apneas). Indeed, hypertension, which is known to increase wall shear stress, is an accepted risk factor for AD^16^ and was present in the large majority of our patients. Genetic factors are also likely to contribute to dissection risk, even aside from diseases of the aortic wall known to be associated with AD (e.g., Ehlers‐Danlos, Marfan Syndrome).

It is also possible that AHI is not the best measure to connect OSA with AD. Recent literature has found that nocturnal hypoxia is strongly associated with cardiovascular risk^17^, ^18^ and ascending aortic size has been reported to be related to the degree of nocturnal hypoxemia.^14^, ^19^ While measures of oxygen desaturation were not investigated in the current study desaturations are a hallmark of OSA and it is conceivable that hypoxia of the aortic wall could contribute to dissection risk.

The limitations of this study should be acknowledged. Most important is the small size of the study sample. In an effort to gather a larger number of subjects, we recruited from four institutions. Aside from being an uncommon disease AD has high early mortality, which also limited our enrollment and highlights the possibility of survivor bias impacting our data.

Treatment of OSA can result in symptomatic relief and can be helpful in the prevention and treatment of hypertension, particularly resistant hypertension.^5^ OSA is also associated with cardiovascular risk in general and treatment might mitigate that risk. While our study found no statistically significant association between OSA and aortic size at the time of dissection, a possible signal of such an association was observed. At the present time, we cannot state that OSA per se is a risk factor for AD but further investigation is warranted.

## CONFLICT OF INTERESTS

Virend K. Somers has served as a consultant for Jazz Pharmaceuticals, Bayer, Respicardia, and Baker Tilly, and is on the Scientific Advisory Board for Sleep Number. The remaining authors declare that there are no conflict of interests.

## Data Availability

Data are available on reasonable request from the authors.
